# Heritage tourism evolution in Guangfu Ancient City, Hebei Province, China: an analysis with the improved creative destruction model

**DOI:** 10.1186/s43238-022-00074-w

**Published:** 2022-10-17

**Authors:** Menghan Wang, Xiaoran Bai, Mingming Su

**Affiliations:** School of Environment & Natural Resources, Renmin University of China, Bejing, 100872 China

**Keywords:** Creative destruction, Creative enhancement, Heritage tourism, Tourism commercialisation, Stakeholder, Guangfu Ancient City

## Abstract

Ancient cities and towns are popular tourism destinations worldwide. In this paper, Guangfu Ancient City in Yongnian County, Hebei Province, China, is taken as the case study and the modified creative destruction model is applied as the analytical framework to evaluate the multiactor dynamics of heritage tourism development. A mixed method approach is adopted, including a local resident survey, in-depth interviews with staff of the Guangfu Ancient City tourism site and government officials responsible for the heritage conservation and tourism development of the site, and a review of online tourist reviews, relevant government documents and reports. Based on the modified creative destruction model, local residents’ attitudes towards tourism development, changes in tourist numbers, the level of business and government investments, and the motivations of different stakeholders in tourism development are assessed by synthesising on-site research, historical data and other materials. Then, the tourism development stages for Guangfu Ancient City are identified as the precommodification stage before 2006, the early commodification stage from 2006 to 2011 and the advanced commodification stage from 2012 to 2017. The findings indicate that with rapid increases in investments from both corporate and government sources and in the number of tourists, the attitude of local residents towards tourism development remained positive. The results show that instead of entering the initial destruction stage, Guangfu Ancient City is in the transition stage from advanced commodification to creative enhancement given the government’s dominant role in tourism development, the heritage conservation motives of tourism entrepreneurs, the benefits to residents from tourism development ensured by government policies, and the shift in tourist type to postmodern tourists with double demands. The applicability of the modified creative destruction model is further discussed, and policy and management recommendations are generated to support the sustainable development of Guangfu Ancient City after the COVID-19 pandemic.

## Introduction

Heritage tourism is a well-analysed area of research and a necessary form of tourism development that is important to most countries and regions that hope to achieve heritage conservation through the tourism use of heritage sites (Fonseca and Ramos 2012; Poria and Ashworth 2009; Schmutz and Elliott 2016). Playing a crucial cultural, educational, political and artistic role, conservation of heritage resources contributes to the social development and cultural preservation of the destination area. However, with the excessive expansion of tourism activities, heritage resources are undergoing a process of value reevaluation and commercial packaging, which may destroy their original landscape and cultural identity (Yang and Greenop 2020; Zhao 2019; van der Merwe 2016). Such a situation is pertinent for China, which has rich heritage resources and is experiencing rapid tourism development. In such a context, how to balance heritage conservation and tourism development and prevent overcommercialisation of heritage tourism destinations is a critical issue for heritage tourism researchers.

The creative destruction model proposed by Mitchell, Atkinson, and Clark (2001) provides a systematic and evolutionary framework to understand the dynamic multiactor process of tourism development and discuss the impacts of commercialisation and the division of development stages for tourist destinations (Mitchell, Atkinson, and Clark 2001). Originally created and used in Western contexts (Mitchell and Vanderwerf 2010), the creative destruction model has been applied in China to analyse the evolutionary process of tourism development in ancient towns such as Luzhi, Daxu and Yangshuo (Fan, Wall, and Mitchell 2008; Qun, Mitchell, and Wall 2012). Such applications supported its suitability for Chinese heritage sites and in interpreting the dynamic and multiactor development process. Meanwhile, limitations were revealed in terms of stereotyping the heritage tourism development pathway in a changing and dynamic context. To address this limitation, Mitchell (2013) revisited the original model and made critical improvements by adding an option of ‘creative enhancement’ to reflect multiple relations between heritage tourism and local development, which has not yet been verified through case studies other than in Canada. In addition, most case studies have been conducted in the southern part of China and more rural contexts (Sofield and Lia 2011), and heritage tourism development in the northern part of China, with relatively different cultural and governance contexts, particularly in urban areas and from evolutionary perspectives, is underrepresented in previous research.

To address the abovementioned research gaps, Guangfu Ancient City in Hebei Province, China, was selected as a case study due to its exemplary value as a heritage town involved in tourism in the northern part of China. The revised creative destruction model was applied to examine the multiactor dynamics of heritage tourism development, which could support future applications and adaptations of this model to heritage sites in China. A mixed method approach was adopted, including a questionnaire survey of local residents of Guangfu Ancient City and in-depth interviews with government officials for heritage conservation and tourism development and the staff of the responsible tourism operation agency. This study contributes to the understanding of the multiactor heritage tourism development process and identifies opportunities and constraints for further sustainable development of heritage towns. In particular, by incorporating the attitudes and perceptions of local residents, this study complements previous studies of the tourism evolution of heritage towns that have mainly emphasised macrolevel perspectives. Additionally, this study proposes recommendations to promote sustainable heritage tourism development in Guangfu Ancient City.

## Literature review

### Heritage tourism in ancient cities and towns

With rich historical and cultural resources that can satisfy tourists’ feeling of nostalgia, ancient cities and towns have been used for tourism throughout the world (Timothy 2003; Wall and Mathieson 2006; Wu, Zhang, and Qiu 2017; Yu and Peng 2015; Su 2019; Su et al. 2020, 2021). With widely recognised positive impacts on supporting heritage preservation and community development (Wu, Zhang, and Qiu 2017; Yu and Peng 2015), tourism in ancient cities and towns can also promote local culture and reinforce residents’ identity (Su and Wall 2019). Moreover, intangible cultural heritage resources, such as traditional lifestyles and cultural expressions, play important roles in constructing meaningful tourist experiences (Su 2019; Su et al. 2020).

With its long history and rich culture, China has a wide diversity of ancient cities and towns, many of which are already popular tourism destinations, such as Lijiang Ancient Town (McCartney and Chen 2020; Su 2019; Su et al. 2020; Wu, Zhang, and Qiu 2017) and Xidi and Hongcun in Anhui Province (Zhang and Smith 2019). Both received United Nations Educational, Scientific, and Cultural Organisation (UNESCO) world heritage designations, indicating that the meanings and cultural significance of such ancient towns and cities, though locally oriented, can be universally understood and valued.

The rapid tourism development of ancient cities or towns in China has led to rich discussions of how to balance heritage preservation and tourism development (Wu, Zhang, and Qiu 2017; Yu and Peng 2015), as well as how to harmonise tourist uses and resident needs (Liu, Pan, and Zhou 2005; McCartney and Chen 2020; Su and Wall 2017). However, excessive tourism uses may hinder local uses and challenge the conservation of such heritage resources, and tensions between commodification and authenticity have been recognised (Liu, Pan, and Zhou 2005; Su 2019; Su et al. 2020; Fan, Wall, and Mitchell 2008; Huang, Wall, and Mitchell 2007). The dynamic interactions of multiple actors through the process of tourism development deserve more research in different geographical and cultural contexts.

### Heritage tourism evolution: creative destruction or creative enhancement

Created by Schumpeter (1942), the concept of ‘creative destruction’ was initially an attempt to integrate the process of creation and destruction into the production cycle and was traditionally used to explain changes in the secondary and primary sectors. Adapting this concept to the field of geography, Harvey (1985) believed that entrepreneurial investments not only drove the production cycle but also played an important role in facilitating the creation and destruction of landscapes (Mitchell 1998). Thus, the creative destruction model can also be applied to the study of cultural development in the context of globalisation (Harvey 1987, 1988; Harvey and Mackinder 1990).

Based on the above perspectives, Mitchell proposed the model of creative destruction that was initially applied to heritage communities in the village of St. Jacobs, Ontario, Canada (Mitchell and de Waal 2009). The model has three prerequisites: an attractive cultural landscape, a large city in close proximity providing a sufficient source of tourists, and some drivers of commercial development in the area (Mitchell and de Waal 2009). Encompassing six stages distinguished by changes in a number of variables, as shown in Table 1, the model describes changes occurring in the community at different stages in terms of relationships between business investment, number of tourists and residents’ attitudes towards tourism development (Huang, Wall, and Mitchell 2007). The argument is that when a new kind of landscape is created through investment, the old is simultaneously destroyed (Mitchell and de Waal 2009). Mitchell’s stage model indicates that three rational landscapes, the purpose-scape, the heritage-scape, and the leisure-scape, will be created and sequentially destroyed over time.

**Table 1:** The six-stage model of creative destruction

Stage	Activities of drivers: profiteers, preservationists, and promoters	Consumers (hosts and guests)	Attitudes towards tourism	Dominant landscape
Precommodification	Inactive	Few	Largely positive	Productivist
Early commodification	Private-sector investments in commodification are initiated. Preservationist activity may be initiated. Policy promoting development may be implemented.	Some heritage seekers	Some awareness of negative implications	
Advanced commodification	Active private-sector investments in commodification.	Growing numbers of heritage seekers	Increasing awareness of negative implications	Postproductivist heritage-scape
Early destruction	Very active private-sector investments. Some will deviate from the heritage theme. Preservationists may actively oppose nonheritage investments (often unsuccessfully). Public-sector policy/action promoting development may be implemented or continue.	Heritage seekers accompanied by postmodern tourists.	Much awareness of negative implications.	
Advanced destruction	Scale of private-sector investment increases (e.g., hotels), with much deviation from the heritage theme. Preservationists may actively oppose nonheritage investments (often successfully). Prodevelopment policies/actions may be implemented or continue.	Postmodern tourists are in the majority.	The majority of residents offer negative comments; outmigration may occur.	
Postdestruction	Nonheritage, private-sector investments dominate. Preservationist activity may be diminished. Prodevelopment policies may be in place.	Number of heritage seekers is very low.	The overall attitude in the community is positive, and few negative attitudes remain.	Neoproductivist leisure-scape

Source: Based on Mitchell’s six-stage creative destruction model (Mitchell and de Waal 2009)

Following Mitchell’s early work, the creative destruction model was subsequently applied to different sites in Canada (Mitchell and Vanderwerf 2010) and to heritage towns in North America (Hetzler 2000; Tonts and Greive 2002), China (Qun, Mitchell, and Wall 2012; Huang, Wall, and Mitchell 2007; Fan, Wall, and Mitchell 2008), Australia (Tonts and Greive 2002) and Japan (Chang, Su, and Chang 2011). Despite some disagreements on the existence of landscape substitution (Mitchell and Sullivan 2012; Shannon and Mitchell 2012), previous studies supported the existence of evolutionary stages at heritage tourism destinations, and the creative destruction model can effectively be used to examine degrees of commercialisation and explore the impacts of tourism investments at heritage sites. However, precious research in China, where tourism development usually has a strong government influence compared with a more market-driven mode in the Western context, also revealed restrictions of the creative destruction model in understanding the tourism development process at heritage towns or villages in China; in particular, there were some debates about features that define the destruction phases (Qun, Mitchell, and Wall 2012; Huang, Wall, and Mitchell 2007; Fan, Wall, and Mitchell 2008).

**Fig. 1:**
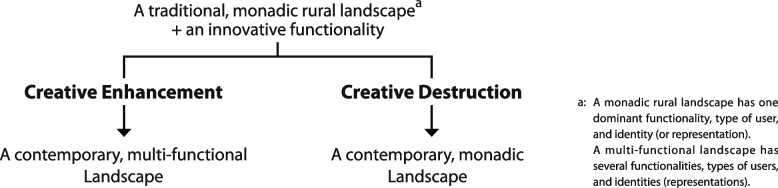
Processes of change in a rural space (Source: adapted from Mitchell 2013)

To address restrictions on applying the creative destruction model in different contexts and following her continuous study of small heritage towns in Ontario, Canada, Mitchell (2013) proposed the addition of a ‘creative enhancement’ phase to the original creative destruction model. This is a harmonious multifunctional phase in which new landscapes and functions are created along with original landscapes and functions being maintained, indicating a shift from the production-based economy to a multifunctional economy in some villages. As shown in Fig. 1, the innovative coexistence of the new and the original indicates an alternative pathway of heritage destinations through tourism development, which supports the sustainable development of heritage communities with various conserved space functions to satisfy the needs of both tourists and residents.

Mitchell (2013) argued that if the tourist demand for entertainment and merchandise is low, features of original functions are more likely to be retained to meet residents’ needs. Moreover, the closer the spatial proximity between new and original functions, the greater the likelihood of function coexistence. To facilitate the coexistence of multiple landscapes and functions to achieve the phase of creative enhancement, the attitudes and roles of stakeholders, such as local governments, residents, and tourism enterprises, are also critical.

Accumulated over years of heritage tourism practices and research, Mitchell’s improved creative destructive model (2013) further reflected the dynamic relations between heritage and tourism. By proposing the potential stage in which the heritage space can coexist with the tourism space to satisfy both residents and tourists, it echoes trends to facilitate the integration of tourism with other industries and needs for the sustainability of heritage destinations. Therefore, the improved creative destructive model fits better with the evolution of research in heritage tourism. Thus, it is chosen as the framework to guide the analysis of the heritage tourism evolution of Guangfu Ancient City.

## Research methodology

### Study sites

**Fig. 2:**
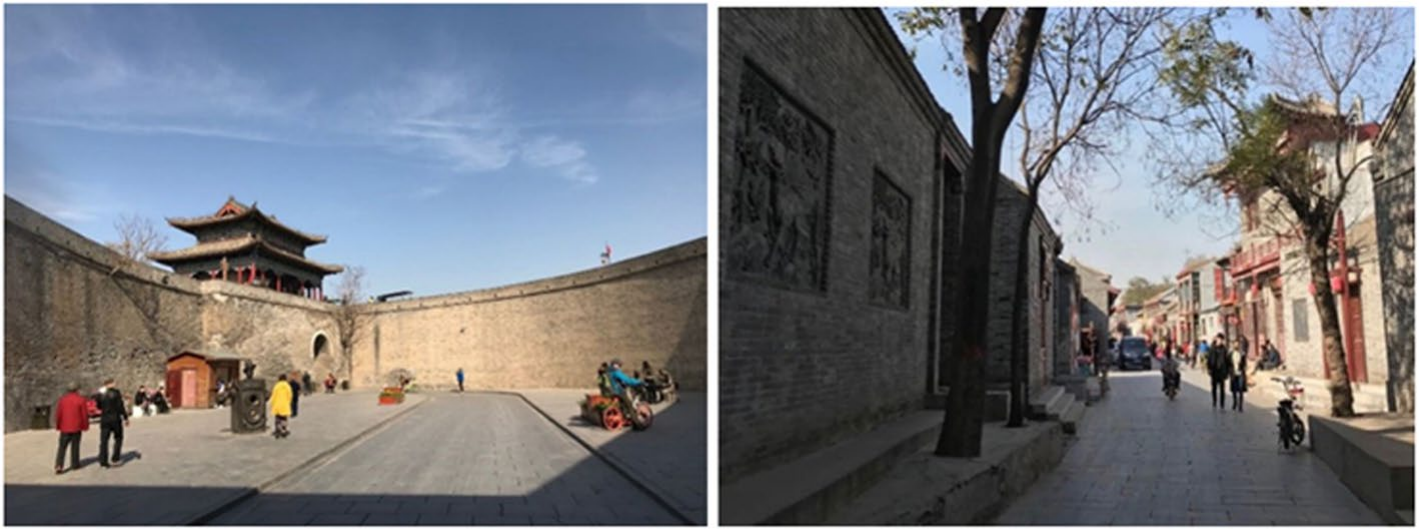
Examples of Guangfu Ancient City, Hebei Province (Source: Mingming Su)

With more than 2.6 thousand years of history, Guangfu Ancient City is known for its well-preserved city wall and ancient urban pattern that originated in the Tang Dynasty and was reconstructed in the Ming Dynasty (Fig. 2). With a total area of 1.5 km^2^, Guangfu Ancient City hosts 7149 local residents (Tao and Zhang 2021). There are two national key cultural heritage sites and more than 20 provincial and municipal cultural heritage sites in the city. Guangfu Ancient City is also known as the hometown of Tai Ji, as the founders of Yang’s and Wu’s Tai Ji practices were born there. Owing to its historical and cultural importance, Guangfu Ancient City was designated a 5A National Tourist Attraction in 2017.

**Fig. 3:**
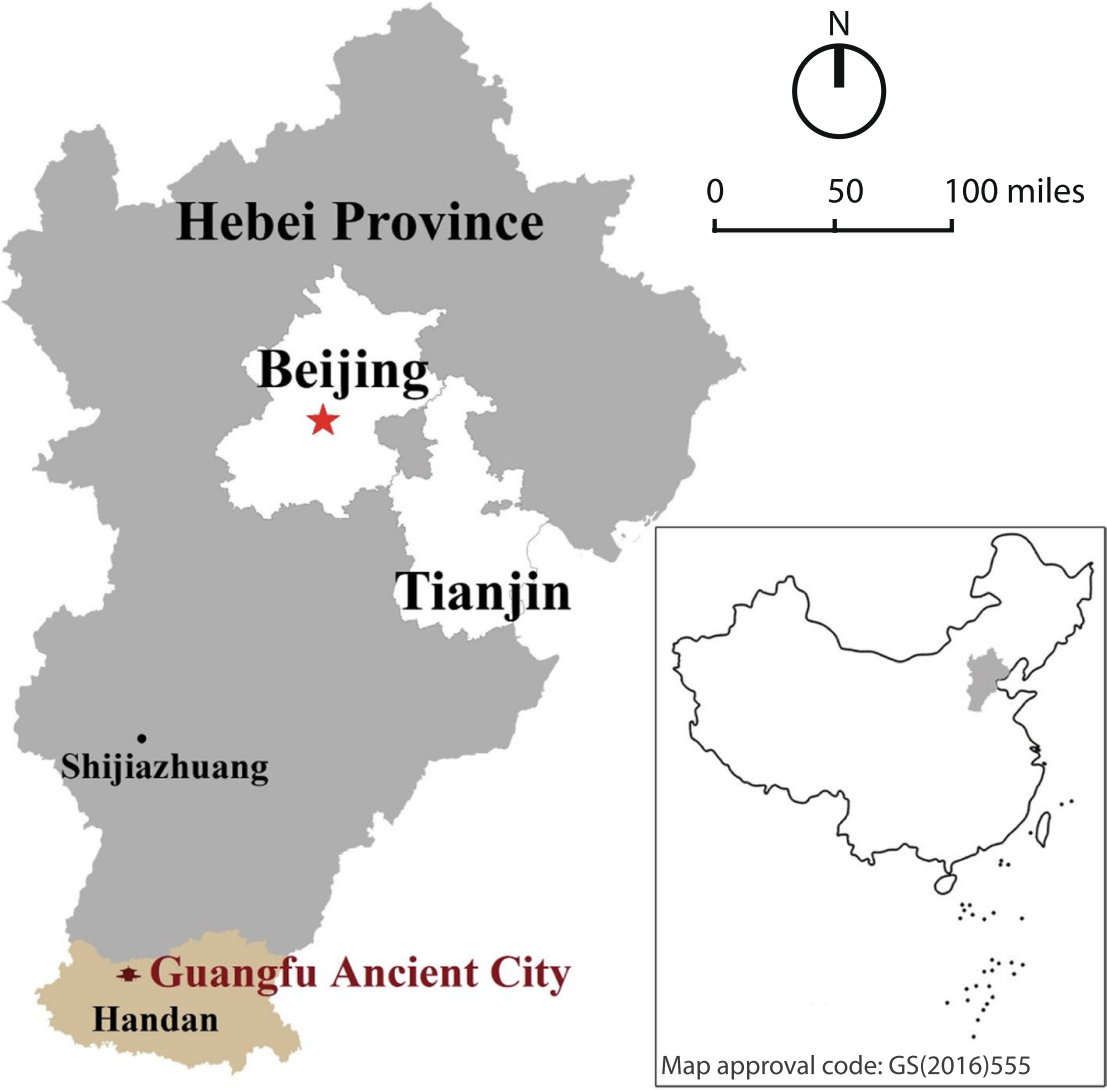
The location of Guangfu Ancient City, Hebei Province, China (Source: the authors)

Conveniently located in southern Hebei Province, Guangfu Ancient City is only 15 km from Handan city to the southwest; 160 km from Shijiazhuang city, the capital of Hebei Province, to the north; and 420 km from Beijing, the capital of China, to the north (Fig. 3). Surrounded by major transportation infrastructure, such as Handan Airport, Beijing-Guangzhou Railway, Shi-Wu Expressway Railway and National Highway 107, Guangfu Ancient City is also highly accessible for tourists from nearby major cities.

Recognising the importance of Guangfu Ancient City’s cultural heritage and potential for tourism, both public and private initiatives were deployed for heritage tourism, particularly after 2006, which drew investment and drove tourism development on a fast track. According to the Yongnian District Government Report, total tourism receipts reached 1.853 million with tourism revenue of 0.45 billion RMB in 2019, and the accumulated investment reached 1 billion RMB in 2019 (Tao and Zhang 2021).

As it meets all three criteria to engage the improved creative destruction model for analysis (Mitchell and de Waal 2009), Guangfu Ancient City is a suitable case study for the evolutionary analysis of heritage tourism destinations.

### Research methods

Data collection was based on the three variables that drive the creative destruction process, integrating key informant interviews, the questionnaire survey of residents, field observations and a review of government reports and documents. Data collected from different sources were triangulated to support the analysis of the heritage tourism evolution process.

During the first period of field research from October 21 to October 25, 2017, initial contacts were made with government officials, and basic information on the tourism development of Guangfu Ancient City was collected. Additionally, a pretest of the resident questionnaire survey was conducted with local residents. Revisions to the questionnaire and semistructured interview questions were made accordingly. The formal field research was conducted from November 7 to 14, 2017, when the formal resident questionnaire was distributed and the semistructured interviews were conducted.

The resident questionnaire survey was distributed in Guangfu Ancient City in November 2017. Resident attitudes towards tourism development from economic, environmental and sociocultural perspectives were measured on a 5-point Likert scale. Residents were invited randomly on the streets, and the research aims were explained to the respondents. On average, each questionnaire took approximately 10 min to complete, and 71 valid questionnaires were collected of 100 distributed. Of the respondents, 52% were men and 48% were women.

In-depth and face-to-face interviews with key informants from the local government and key management organisations of Guangfu Ancient City (Table 2) were conducted in October and November 2017 with questions on the overall development of Guangfu Ancient City, government policy and development strategy, level of private investment, type and scale of tourists, and involvement and attitudes of residents. A 20-min follow-up interview with the staff of the Guangfu Ancient City tourism site was conducted through a WeChat call in May 2022 to acquire additional information about recent tourism development and tourism statistics, including mainly tourist arrivals, tourism revenue, and numbers of restaurants and accommodation providers from 2012 to 2019. The interviewed staff commented,

*‘Due to the government’s continuous infrastructure improvements, tourism in Guangfu Ancient City has been further developed from 2012 to 2019; in particular, the number of restaurants and accommodation providers is gradually increasing, which has attracted many tourists and investors.’*

**Table 2:** List of interviewees and documents reviewed

Key informants interviewed	Duration and form of interview
✓ Director of the establishment office of Guangfu Tourism Management Committee	40 min, face to face
✓ Team leader of Guangfu 5A Office	30 min, face to face
✓ Deputy director of Guangfu Management Committee	30 min, face to face
✓ Staff of Guangfu Ancient City tourism site	20 min, WeChat
Main government documents reviewed	
✓ Introduction to Guangfu Ancient City Cultural Tourism Development Project	
✓ Summary of work to designate Guangfu as a National 5A Tourism Destination	
✓ Summary of Guangfu Ancient City tourism development, 2008–2017	
✓ Tourism statistics, 2012–2019	

In addition to the resident survey and key informant interviews, secondary data, mainly government documents and reports related to local tourism development (Table 2), were collected and reviewed. Tourist online reviews of Guangfu Ancient City were retrieved from the Mafengwo online travel platform (Mafengwo.com) to assist in developing an understanding of tourist behaviour and experiences. A total of 153 tourist reviews comprising 9704 Chinese characters and posted between 2012 and 2019 were obtained for content analysis. In addition, the third author’s personal observations of tourism development over the years and field notes during the field research were used as supplemental information.

## Stages of creative destruction of Guangfu Ancient City

Based on the improved creative destruction model and data collected from different sources, the stages of heritage tourism development in Guangfu Ancient City were classified into stages of precommodification (before 2006), early commodification (2006 to 2011) and advanced commodification (after 2011). The dynamics of the advanced commodification stage were examined in detail from a multiactor perspective.

### Precommodification stage

According to the interviews with government officials and managers, prior to the restoration of the ancient city wall in 2006, Guangfu was under the management of the town government as a section-level unit of Yongnian County. Local development was dominated by small businesses and fishing. The recollections of local residents and interviewees indicated that tourism resources were not yet developed, and historical sites were unexplored. Although the idea of tourism development did not emerge in this period, there were sporadic visits by photographers and history lovers who came to visit Guangfu Ancient City. This created the necessary conditions for tourism investments in Guangfu Ancient City to occur in the next stage.

### Early commodification stage

The stage of early commodification started in 2006, when public and private investments became the main driver of tourism development, which was supported through the development of the Guangfu Ancient City tourism plan, institutional rearrangements for tourism management and targeted marketing and product development.

The interviews and document review revealed that following the initial small-scale restoration of Guangfu Ancient City, funded by the chair of the Silicon Valley Company in Yongnian County, the official restoration of the 4522-m-long Guangfu Ancient City wall was led by the local government and has been invested in by the same company since June 28, 2006. Moreover, the local government invited relevant planners and experts to develop the *Guangfu Ancient City Protection and Restoration Plan* in 2007. To facilitate tourism management, the state-owned Guangfu Tourism Development Company was established in 2008 by the local government. Using the company’s platform, the government organised a number of investment activities and invited large companies to visit the restored Guangfu Ancient City. A number of tourism development projects were successfully initiated, indicating that the development of Guangfu had begun to move towards a market-oriented development path.

In 2011, the government implemented a comprehensive renovation project to restore the historical appearance and improve the infrastructure and physical environment of Guangfu Ancient City. However, due to financial limitations and the overall small scale of investments, the basic infrastructure of Guangfu Ancient City during that period was still weak, restricting the further expansion of investments. An interviewed government official commented,

*‘At the beginning of tourism development, Guangfu Ancient City faced great difficulties in attracting investments with weak infrastructure. We needed to further improve the infrastructure in order to attract more investments.’*

To increase the number of visitors, the local government implemented promotional activities in targeted tourism markets, particularly in nearby cities, such as Handan, Anyang and Xingtai. In addition, packaged tours from Beijing and Shijiazhuang to Guangfu Ancient City were organised through cooperation with travel agencies. Moreover, journalists from central, provincial and municipal news media were invited to Guangfu Ancient City to increase its media exposure. In 2010, the TV series ‘The Legend of Guangfu Tai Ji’ was broadcast on CCTV to enhance the popularity of Guangfu Ancient City. According to official statistics released by the management committee, the number of visitors increased by 2.7 times, from 150,000 in 2007 to 550,000 in 2011. According to interviews with local residents, they generally held supportive attitudes towards tourism development due to the improvements to the infrastructure and environment resulting from tourism development,

At this stage, investments by the local government and private companies in combination drove the process of heritage conservation and tourism development (Table 3). Guangfu Ancient City was developed into an emerging heritage tourism attraction with the creation of the initial heritage landscape. With a growing number of tourists, private entrepreneurs began to construct entertainment facilities. The commercial entertainment landscape began to emerge.

**Table 3:** Major investments made during the early commodification stage

Investments	Progress	Investment amount
**Projects of the heritage landscape**		
Hongji Bridge Park expansion project	Planning	55 million yuan
Qinghui Academy and Hotel construction project	Planning	160 million yuan
Street improvement project	Start to promote	20 million yuan
Node creation project in the city	Start to promote	15 million yuan
Relocation project in Yangling	Start to promote	10 million yuan
Agricultural tourism park project	Completed	
**Projects of the recreational landscape**		
Comprehensive development project of reed swamp	Planning	10 million yuan
Tai Ji ecological manor	Planning	350 million yuan
Water tourism corridor around the city	Planning	45 million yuan

Data Source: Summary of Guangfu Ancient City tourism development, 2008–2017

### Advanced commodification stage

A rapid increase in investments marked the entry of Guangfu Ancient City into the state of advanced commodification in 2012. Driven by large-scale investments from both the government and private enterprises, Guangfu Ancient City experienced noticeable changes in its physical landscape and a drastic increase in both the number of tourists and tourism revenue.

Public investments were used for a series of projects for heritage restoration and infrastructure enhancement. As indicated in government reports from 2014 to 2017, the local government invested 600 million RMB to support the National 5A Tourism Destination application of Guangfu Ancient City (the highest rank in the Chinese official rating of destinations). The ancient city wall and the city gate tower were restored, and the former residences of Yang Lu Chan and Wu Yuxiang were fully repaired. In addition to heritage conservation and restoration, the local government invested 1 billion RMB in infrastructure development, including the renovation of the surface of Guangfu Ancient City, from 2015 to 2017. The results of such investments were successful, as Guangfu Ancient City was approved as a National 5A Tourism Destination by the Chinese National Tourism Administration in 2017.

Private investments from enterprises were used mainly for the modernisation of tourist attractions and the construction of recreational facilities. As shown in Table 4, key investments in heritage conservation and renovation projects reached approximately 900 million RMB, and projects for recreational facilities accumulated to approximately 3500 million RMB during this period. Such an increase in investments changed the heritage and tourism landscapes of Guangfu Ancient City and drove its transformation into a tourism space.

**Table 4:** Major investments during the advanced commodification stage

Investments	Progress	Investment (million RMB)
**Projects of the heritage landscape**		
‘Wu’s Courtyard’ renovation	Completed (2014)	15
Expansion of Wu Yuxiang’s former residence	N/A	35
‘Tong’s Courtyard’ restoration	Completed (2016)	40
Restoration of old city government	Completed (2016)	85
Qinghui Academy and International Tai Ji Exchange Centre	In progress	160
Construction of the ancient street of South Street	Planning	200
Zhao’s restaurant restoration	Completed	40
Node renovation and upgrading project	In progress	143
Tai Ji Cultural Industrial Park	Planning	150
Tai Ji themed performance	Planning	5
Tai Ji culture and event performance	Planning	9.86
**Projects of the tourism landscape**		
Xiguan Waterpark Project	Completed (2014)	120
Water Reception Centre	Completed (2014)	50
Tai Ji City	In progress	3000
Tai Ji Code Water City	In progress	300
National Angling Centre project	In progress	30

Data Source: Summary of Guangfu Ancient City tourism development, 2008–2017

In addition to large-scale development, a growing number of small-scale private investors joined the tourism development of Guangfu Ancient City through the operation of different types of retail stores. Field observation revealed that the retail stores concentrated in South Street, East Street and Dong Guan of Guangfu Ancient City could be classified into three categories: local snack shops or restaurants, handicrafts and antiques shops and accommodations. Through interviews with Guangfu Ancient City management staff, substantial growth in restaurants and accommodation providers was recorded in Guangfu Ancient City from 2012 on.

**Fig. 4:**
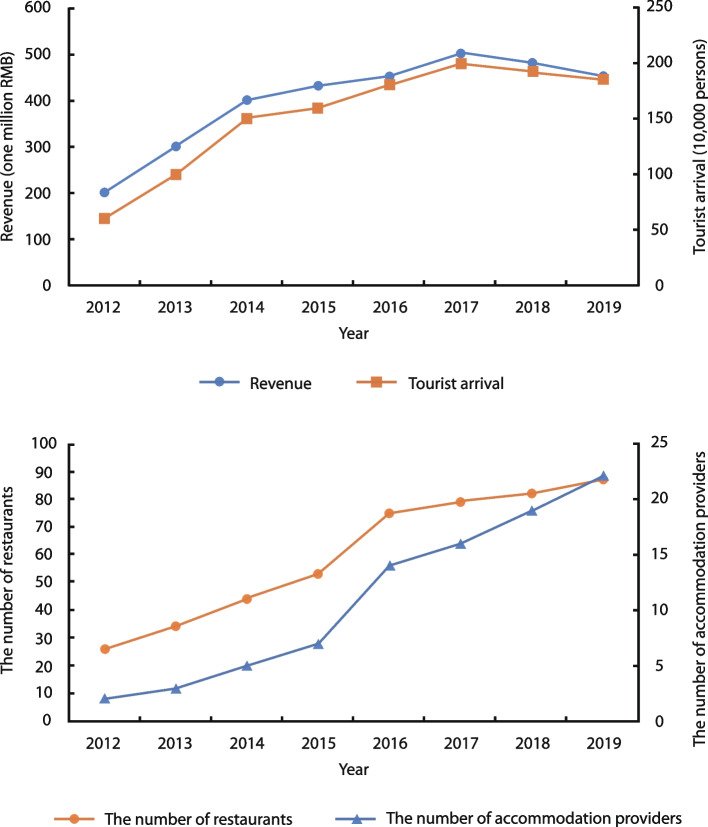
Increased tourist arrivals and revenue (top) and businesses (bottom) in Guangfu Ancient City (Source: the authors)

As a result of both public and private investments, the number of tourists tripled from 0.6 million in 2012 to 1.85 million in 2019. Comprehensive tourism revenue also climbed from 200 million RMB in 2012 to 450 million RMB in 2019 (Fig. 4). According to interviews and field observations, tourists were attracted to Guangfu Ancient City due mainly to local heritage resources and entertainment facilities. As indicated by the Mafengwo online travel website, the total number of tourist reviews of Guangfu Ancient City reached 120 from October 2013 to February 2018. Among these reviews, 58 mentioned only historical buildings and traditional culture, 9 specifically discussed entertainment projects, and 39 covered both topics. In addition, as indicated in the management interviews, a large number of domestic and foreign tourists were attracted by the opportunity to experience Guangfu Tai Ji culture and learn Tai Ji.

It has been well documented that the presence of tourism activity has implications for the host community (Wang, Zeng, and Zhong 2017), and Guangfu Ancient City was no exception. Residents’ attitudes towards tourism development were evaluated through the resident survey. As shown in Table 5, among 71 valid questionnaires collected, respondents were evenly distributed in terms of gender, while the majority of respondents were aged between 28 and 37 years.

**Table 5:** Sample description of Guangfu Ancient City residents (71 valid questionnaires)

Variable	Number	Percentage
**Age**		
18–27	11	15.49
28–27	30	43.25
38–47	22	30.99
48–57	6	8.45
58+	2	2.82
**Gender**		
Female	37	52.11
Male	34	47.89

Residents were asked to evaluate the economic, environmental, and social impacts of tourism (Table 6). The majority of the respondents agreed that tourism had improved their personal income, employment opportunities and the standard of living. However, prices of goods and land were perceived as having increased as a result of tourism (Mean = 3.775). Although some negative evaluations were found from some respondents, indicating that tourism increased local traffic (Mean = 3.662) and created noise pollution (Mean = 3.014), particularly during major festivals and holidays, the evaluation of environmental impacts was generally positive, particularly for local historical and cultural heritage conservation (Mean = 4.127), beautifying the living environment (Mean = 4.056) and improving infrastructure (Mean = 3.930). This evaluation was also confirmed by the low agreement (Mean = 1.5) with the statement ‘Tourism development has brought inconvenience to my life.’ Moreover, the respondents’ overall evaluation of the sociocultural impacts of tourism were generally positive, such as raising the awareness of Guangfu Ancient City (Mean = 4.3), preserving Guangfu Ancient City (Mean = 4.2) and enhancing local pride (Mean = 4.2).

**Table 6:** Residents’ perceptions of the impacts of tourism

Statement	Mean score	Standard deviation
**Economic**		
Improves personal income	4.042	0.516
Increases employment opportunities	3.958	0.568
Improves residents’ living standard	3.930	0.454
Increases prices of land and commodities	3.775	0.481
**Environmental**		
Protects historic and cultural heritage	4.127	0.626
Beautifies the living environment	4.056	0.603
Improves public infrastructure	3.930	0.539
Leads to traffic jams	3.662	0.649
Leads to overcrowding	2.831	0.964
Increases noise pollution	3.014	0.957
Leads to inconvenience	1.535	0.689
Increases public safety issues	3.310	0.849
**Sociocultural**		
Raises awareness of Guangfu Ancient City	4.254	0.549
Preserves Guangfu Ancient City	4.183	0.589
I am proud to be a resident of Guangfu	4.211	0.472
**Overall**		
Positive impacts of developing tourism outweigh negative impacts	3.775	0.735
I support further tourism development in Guangfu	3.872	1.048

Although some negative impacts were recognised, the resident survey revealed general support for further heritage tourism development, and the current level of positive impacts received from tourism outweighed the negative impacts. The increase in business investment by enterprises has not led to a reduction in government investment in local infrastructure expansion, street renovation, monument maintenance, and environmental optimisation. For example, the ‘toilet revolution’ in 2016 as well as the annual renovation and restoration of ancient buildings such as Wu Yuxiang’s former residence, Wu’s Courtyard, and Tong’s Courtyard have led local residents to believe that tourism development has brought convenience and environmental improvement. As one government official noted,

*‘The government mainly relied on the entrance fee; tourism provided opportunities for local residents to participate in many other types of tourism-related businesses, such as souvenirs, catering, accommodation, etc. The government also renovated several houses as signature stores or hotels for residents to operate.’*

**Fig. 5:**
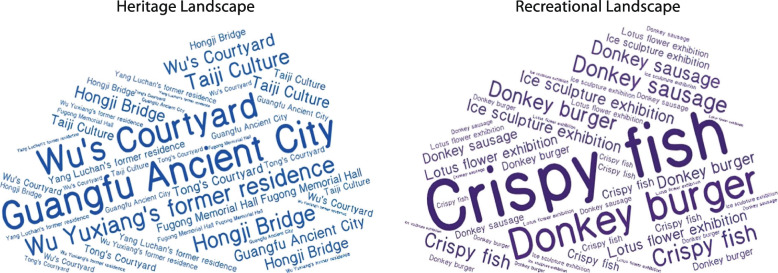
Word cloud analysis of tourist reviews of Guangfu Ancient City (Source: the authors)

In addition, tourist reviews on the Mafengwo website, one of the top travel websites in China, were collected and reviewed. Word cloud analysis was performed, as shown in Fig. 5. Key heritage landscapes recognised by tourists were Wu’s Courtyard, the former residence of Wu Yuxiang, Hongji Bridge and Tai Ji culture. Key recreational landscapes recognised by tourists included mainly local signature foods, such as donkey meat burgers and sausages, and local recreational activities, such as lotus and ice sculpture exhibitions. An evident increase in the number of tourist reviews had occurred since 2017 (Table 7) in response to the approval of the National 5A designation and supporting tourism development. In comparison with recreational landscapes, a gradual increase in tourist attention to heritage landscapes was noted, with a total of 67.4% of tourist reviews mentioning heritage landscapes in 2019, including 47.5% mentioning heritage landscapes only and 20.0% mentioning both heritage and recreational landscapes.

**Table 7:** Tourist reviews of Guangfu Ancient City from Mafengwo travel website

Year	Total reviews	Number of reviews mentioning heritage landscapes	Number of reviews mentioning recreational landscapes	Number of reviews mentioning both heritage and recreational landscapes	Number of Chinese characters
2012–2015	12	41.6%	16.7%	8.3%	1040
2016	19	42.1%	10.5%	21.1%	1033
2017	36	38.9%	16.7%	22.2%	2329
2018	46	45.7%	19.6%	15.2%	2899
2019	40	47.5%	12.5%	20.0%	2403

Tourist reviews started to appear in 2012, but the number of reviews prior to 2016 was small

## Development stages of heritage tourism in Guangfu Ancient City

Based on the improved creative destruction model proposed by Mitchell (2013), the progress of heritage tourism development of Guangfu Ancient City was examined in this study. Consistent with previous studies (Huang, Wall, and Mitchell 2007), the findings indicate that driving forces, the type and number of tourists, and attitudes of local residents played critical roles in the tourism development process of Guangfu Ancient City. Both public and private investments served as the main drivers in the commodification of heritage resources for tourism use. As a result, the number of tourists and the level of tourism revenue continued to grow, which also reinforced further investments on a larger scale. Receiving more benefits than costs, local residents generally held positive attitudes towards tourism development.

**Fig. 6:**
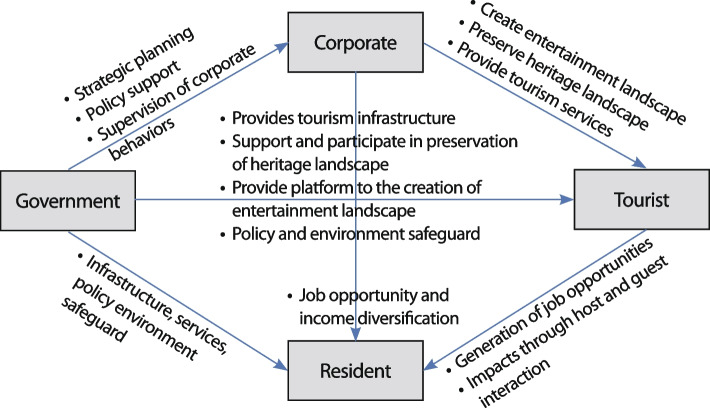
Multiple stakeholder interactions in Guangfu Ancient City (Source: the authors)

The interactions of four key stakeholders throughout the development process of heritage tourism in Guangfu Ancient City are presented in Fig. 6. Through policy making, planning, and direct and indirect investments, the local government played a critical role in the preservation, promotion, production and commodification of heritage landscapes, which yielded financial gains for residents and helped Guangfu Ancient City to become a national tourism destination. In contrast to the market- or capital-driven development mode in the Western context, the high involvement of governments in heritage tourism development safeguarded the social and cultural functions of heritage resources and the needs of residents and promoted Guangfu Ancient City towards the ‘creative enhancement’ stage of development. As one government official described,

*‘Guangfu Ancient City is an important 5A National Tourist Attraction in Handan, and the local government attaches great importance to the development of tourism in Guangfu Ancient City. The protection and management of the ancient city is now much more regulated than before.’*

Second, the findings indicated that, in contrast to previous studies (Mitchell and de Waal 2009; Mitchell 2013), local entrepreneurs demonstrated high motivation for heritage conservation in addition to the pursuit of profits. Under the influence of Chinese traditional culture, the act of giving personal wealth to one’s hometown and society is considered culturally appropriate and can enhance the personal prestige and social status of the entire family (Wei 2010; Biggs, Hall, and Stoeckl 2012). Thus, it is not uncommon for local entrepreneurs to contribute to the preservation of local heritage landscapes. Such motives can also support the balancing of heritage conservation and tourism development.

Third, as outlined in the creative destruction model, an outmigration of local residents may occur as crowding and congestion become unbearable in the early destruction stage. In contrast, in Guangfu Ancient City, despite the acknowledgements of the existence of negative impacts, residents gave high evaluations to tourism development and expressed a strong attachment to their hometown and increased local pride as a result of the restoration of Guangfu Ancient City and the subsequent tourism development. Therefore, the results did not support the occurrence of early destruction in Guangfu Ancient City.

**Fig. 7:**
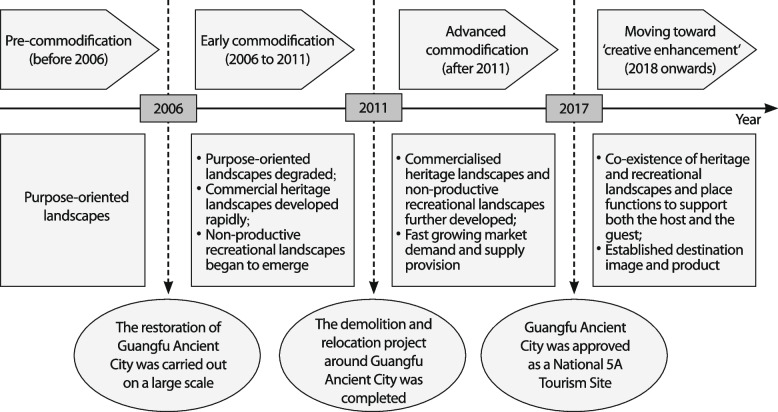
Heritage tourism development stages of Guangfu Ancient City (Source: the authors)

As a result, the first three development phases of Guangfu Ancient City were in accordance with the original creative destruction model. Instead of moving towards destruction stages, as outlined in the original creative destruction model, Guangfu Ancient City is in the transition phase from ‘advanced modification’ to ‘creative enhancement’, with traditional landscapes and functions maintained along with the development of the new (Fig. 7). Key reasons include the government’s dominant role in tourism development, the heritage conservation motives of tourism entrepreneurs, the positive attitudes and enhanced local pride of residents from tourism development ensured by government policies, and the shift in tourist type to postmodern tourists with double demands for both heritage and tourism landscapes.

However, the development of Guangfu Ancient City has inevitably been challenged by the COVID-19 pandemic since 2020. As revealed by the interviewed management staff, the site operation was suspended for most of the pandemic, which substantially undermined its further development. Therefore, whether a ‘creative enhancement’ phase can be reached is also subject to the extent to which tourism may recover after the pandemic and how site management can react to such changes, along with the support and collaboration of other actors.

## Conclusion

With the case of Guangfu Ancient City, this study further discusses the improved creative destruction model (Mitchell 2013), in which a ‘creative enhancement’ stage was introduced with multiple landscapes and functions coexisting to support both host and guest. The results illustrate that the improved creative destruction model with possible pathways towards either ‘destruction’ or ‘enhancement’ shows higher feasibility in explaining the dynamic multiactor evolutionary process of heritage tourism destinations, analysing drivers for development and identifying pathways to ensure the sustainability of heritage destinations. Future studies could further extend the model to other heritage tourism destinations in China and in other contexts.

For Guangfu Ancient City, considering levels of investment, changes in landscapes and space functions, tourist characteristics and residents’ attitudes, three development stages were identified: the precommodification stage before 2006, the early commodification stage from 2006 to 2011 and the advanced commodification stage from 2012 to 2017. For the post-2017 phase, the increase in both tourist arrivals and revenues flattened along with the level of investments (Fig. 4), indicating an established tourism market. Based on the improved creative destruction model, the argument is that instead of moving towards the early destruction stage, Guangfu Ancient City is in the transition phase from advanced commodification to creative enhancement, with both new and traditional landscapes and functions for tourists and residents. The government’s dominant role in tourism development, the heritage conservation motives of local tourism entrepreneurs, the benefits to residents from tourism development ensured by government policies, and the shift in tourist type to postmodern tourists with double demands are key in promoting this movement.

Practical recommendations were put forwards to assist Guangfu Ancient City in moving towards the creative enhancement stage through collaboration among government, enterprises, tourists and local residents. First, the results of the analysis supported the important role of local government in heritage tourism development in Guangfu Ancient City. To further satisfy the diverse needs of both host and guest, it is suggested that municipal tourism plans and urban plans be further developed and integrated to facilitate the coexistence of multifunctional landscapes. Second, with the shift in tourist type from pure sightseers to tourists with mixed motivations, tourist demand for heritage experience and high-quality tourism services and products should be satisfied. Therefore, in addition to enhancing heritage tourism experiences through heritage conservation, interpretation enhancement and experience diversification, it is necessary to formulate and improve the relevant laws and regulations to standardise tourism operations and enhance service qualities to meet tourists’ high demand for entertainment landscapes. Third, despite the current high resident support for tourism indicated in the survey, resident participation in heritage tourism is still restricted in terms of both scale and modes of participation. Further resident participation mechanisms should be developed to enhance resident benefit sharing and participation in planning and decision making to ensure that residents’ interests are well represented. With the ancient city shifting from residents’ living space to a space shared by residents and tourists, residents’ needs should be heard and satisfied. Finally, with the shifts in the landscapes and functions of Guangfu Ancient City, the needs and expectations of tourists will change accordingly. To better understand the dynamics of tourist demands, regular tourist surveys should be conducted, and relevant adjustments to product design and service provision can be made accordingly.

However, with the COVID-19 pandemic posing drastic threats to tourism globally, Guangfu Ancient City is facing serious challenges, with tourism having been suspended from time to time since 2020, which has interfered with its evolution process and led to uncertainties regarding further development. It is critical to consider measures to respond to the vulnerability of tourism to risks on different scales and to enhance destination resilience to facilitate postpandemic recovery and the sustainable development of heritage tourism in Guangfu Ancient City. Further research after the pandemic is needed to understand the influences of external risks on heritage tourism destination evolution.

Taking an exploratory approach, this study obtained firsthand data from the local government and residents. However, limited by time and resources, the perspectives of tourists and the tourism industry were illustrated only through secondary sources, which restricts the richness and depth of the understanding of tourist and tourist business perspectives. Moreover, the one-off resident survey was limited in number and may represent resident attitudes only at the time of research. Future research could take a longitudinal rather than a cross-sectional approach to follow the dynamic and multiactor process of heritage tourism development in Guangfu Ancient City. In addition, the improved creative destruction model could be further explored in other heritage destinations in China and elsewhere. The definition and measurement of the creative enhancement stage of multiple functions and landscapes can then be detailed, and how such theoretical discussions could support heritage tourism practices can be further discussed.

## Data Availability

The datasets used and/or analysed during the current study are available from the corresponding author on reasonable request.

## Abbreviation

UNESCO: United Nations Educational, Scientific, and Cultural Organisation
